# Evaluation of Dietary Essential Amino Acid Supplementation on Growth, Digestive Capacity, Antioxidant, and Intestine Health of the Juvenile Redclaw Crayfish, *Cherax quadricarinatus*

**DOI:** 10.1155/2024/8767751

**Published:** 2024-02-08

**Authors:** Zongzheng Jiang, Dunwei Qian, Zhenye Liang, Sen Wu, Fenglu Han, Chang Xu, Meili Chi, Erchao Li

**Affiliations:** ^1^Key Laboratory of Tropical Hydrobiology and Biotechnology of Hainan Province, Hainan Aquaculture Breeding Engineering Research Center, School of Marine Biology and Aquaculture, Hainan University, Haikou, Hainan 570228, China; ^2^Hainan Provincial Ecological and Environmental Monitoring Centre, 98 Baiju Road, Haikou, Hainan 571126, China; ^3^Key Laboratory of Healthy Freshwater Aquaculture, Ministry of Agriculture and Rural Affairs, Key Laboratory of Freshwater Aquaculture Genetic and Breeding of Zhejiang Province, Zhejiang Institute of Freshwater Fisheries, Huzhou, Zhejiang 313001, China; ^4^Laboratory of Aquaculture Nutrition and Environmental Health, School of Life Science, East China Normal University, 500 Dongchuan Road, Shanghai 200241, China

## Abstract

The present study was an 8-week feeding trial investigating the effects of lysine and threonine supplementation in vegetable-based diets on growth, antioxidative capacity, and gut microbiota of juvenile redclaw crayfish, *Cherax quadricarinatus* (initial weight 11.52 ± 0.23 g). The lysine and threonine were supplemented to formulate five isonitrogenous (37%) and isolipidic (9%) diets containing 0% (control), 0.2% lysine (L0.2), 0.2% threonine (T0.2), 0.4% lysine (L0.4), and 0.4% threonine (T0.4), respectively. Compared to the control, weight gain rate (WGR) and specific growth rate (SGR) of *C. quadricarinatus* significantly increased with increasing dietary lysine and threonine supplementation from 0.2% to 0.4% (*P* < 0.05). Hepatopancreas trypsin activity significantly increased with increasing levels of lysine and threonine in diets (*P* < 0.05). However, the pepsin, lipase, and amylase activities were not affected by dietary levels of lysine and threonine (*P* > 0.05). Compared with the control, crayfish in T0.4 and L0.4 showed significantly higher glutathione peroxidase (GPx) activity (*P* < 0.05), lower alanine aminotransferase (ALT) activity, and lower malondialdehyde (MDA) content (*P* < 0.05). Supplementation with 0.4% lysine significantly changed the composition of the gut microbiota (*P* < 0.05), which showed a significantly increased relative abundance of *Proteobacteria* and decreased *Firmicutes*, *Actinomycetes*, and *Pontomyces* (*P* < 0.05). The PICRUSt analysis demonstrated that the abundance of the metabolism and cellular processes pathways in the L0.4 group were markedly decreased compared with the control (*P* < 0.05). Meanwhile, a tighter interaction of the microbiota community in crayfish was observed in the T0.4 experimental group. In conclusion, these results suggested that dietary supplementation with 0.4% threonine could significantly promote growth and improve microbial health in juvenile *C. quadricarinatus*.

## 1. Introduction

Lack of nutritional information has become the most important factor limiting the development of formulas for aquatic animals. Plant protein has always been the focus of fish meal substitutes in practical diets due to its higher production and lower cost than fish meal [1, 2]. In a previous study, a cottonseed and soybean meal mixture could be used in the formula with a replace ratio of 47.5% without negative effects on *Ictalurus punctatus* [3]. A similar study has shown that 45% of fish meal can be substituted with fermented soybean meal without detrimental effects on growth of juvenile *Larimichthys crocea* [4]. However, several plant proteins sources contain significantly fewer essential amino acids (EAAs) than fish meal [5]. EAAs shortage in plant protein ingredients cause a low nutrient supply in feed, which cannot satisfy the nutritional requirement of cultured aquatic species [6, 7]. 50% of fish meal can be effectively substituted with mixed cottonseed meal and soybean meal (crude protein ratio 1 : 1) in diet for juvenile *C. quadricarinatus*, which also leads to dietary lysine and threonine deficiency [8]. Lysine and threonine are particularly important for the healthy growth of aquatic animals. Previous studies indicated that fish meal replacement by excessive plant materials significantly affects growth performance, intestinal structure, and microflora composition [9–12]. Therefore, the feed nutrients must be balanced and completed to meet the nutritional requirements and improve body health of aquatic animals [13].

Lysine and threonine are generally two of the most important limiting EAAs in the plant-protein ingredients used in aquafeed [1, 6]. Limiting amino acid supplementation in plant-based diets to balance feed nutrition is a common practice in aquaculture [14]. Daily feed intake, weight gain, and protein synthesis were found to be significantly increased in *Oncorhynchus mykiss* fed with lysine supplementation compared to the control on a high levels plant-based diet [5, 15]. Supplementation of methionine in a diet containing 52% soybean meal could maintain the growth of *Silurus meridionalis* [16]. Supplementation of threonine in a plant-based diet could up-regulate muscle growth-related gene expression and regulate the Nrf2 signaling pathway in hybrid catfish to improve muscle growth and antioxidant capacity [17]. Meanwhile, EAA requirements change at different development stages, and the fast-growing aquatic animals require higher levels of amino acids [14]. Thus, considering the positive effects of lysine and threonine in plant-based feed, a balanced amino acid composition in the diets is highly necessary for the growth of animals.

The redclaw crayfish, *Cherax quadricarinatus*, is an emerging importantly economic species with the advantage of omnivorous habits, high nutritional value, easy cultivation, and good flesh quality [18, 19]. However, at present, there is a lack of research on the nutritional impacts of *C. quadricarinatus*, which is an important impediment in developing the special feed. Thus, the object of the current study was to compare and evaluate the effects of the combined use of cottonseed meal and soybean meal with threonine and lysine supplementation on growth, digestive enzyme activity, antioxidant, and gut health of *C. quadricarinatus*. Furthermore, the dietary lysine and threonine requirement for juvenile *C. quadricarinatus* was evaluated.

## 2. Materials and Methods

### 2.1. Experiment Ethics Statement

The current study was approved by the Animal Use and Care Committee, Hainan University, Haikou, China (HNUAUCC-2020-00004).

### 2.2. Experimental Design and Diets Preparation

Fishmeal, soybean meal, and cottonseed meal were used as the main dietary protein sources, and soybean oil was used as a lipid source in all experimental diets. The control group was defined as a diet without supplementation with essential amino acids (EAAs). Based on the control, lysine and threonine were supplemented at the contents of 0.2% (L0.2 and T0.2) or 0.4% (L0.4 and T0.4) to formulate the four treatment diets. Dietary formula and the proximate composition of all diets are presented in Table 1. All raw materials were fully grounded and sieved through a 60-*μ*m mesh sieve and then mixed with soybean oil and further sieved through a 40-*μ*m mesh sieve. The dry materials are mixed with a moderate amount of distilled water and then extruded through a double-helix producer and made into hard pellets with a 2.0 mm diameter. After that, wet pellets were dried in a dry and well-ventilated room and stored at −20°C. The amino acid profile of all diets is displayed in Table 2.

### 2.3. Experimental Management

All crayfish were obtained from an experimental base, Chengmai, Hainan, China, and the acclimation was maintained in a cement tank for 7 days. After that, a total of 400 healthy crayfish (11.52 ± 0.23 g) were randomly allotted to 20 cages with 20 individuals. In each net cage, polyvinyl chloride (PVC) pipes were installed as shelters to minimize disturbances. All crayfish were fed with five diets twice a day (8:00–8:30 and 18:00–18:30) with a ratio of 6% wet body weight. To maintain the fresh water quality, uneaten food and feces were removed by siphon, and then, approximately 60% of the culture water was exchanged every 7 days. The water temperature environment was kept at 25–28°C, and the dissolved O_2_ and pH were maintained at 5.0 mg/L and 7.8–8.2, respectively.

### 2.4. Sample Collection and Growth Evaluation

The crayfish in each replicate were sampled after 8-week trial. After anesthetized in ice bath, the body weight and body length of all individuals in all replicates were measured. Hemolymph from the pericardial cavity of each individual was sampled using 1 mL sterile syringes. After being stored overnight at 4°C, the hemolymph was centrifuged at 3,500 rpm for 10 min at 4°C, and then, the serum was separated into 200 *μ*L tubes and stored at −80°C until use. All crayfish were dissected quickly on ice to obtain hepatopancreas, tail muscle, and midintestine samples and then weighted and frozen for further analysis. At the end of the feeding trial, three crayfish were collected and stored in a refrigerator (−20°C) for measurements of whole crayfish proximate composition.

The growth evaluation was calculated with the following formulas:

(1) $$\begin{array}{c} Survival\left(\%\right)=100\times \left(Final\;individual\;number/Initial\;individual\;number\right), \end{array}$$

(2) $$\begin{array}{c} Weight\;gain\;\left(WG,\%\right)=100\times \left(Final\;individual\;weight-initial\;individual\;weight\right)/Initial\;individual\;weight, \end{array}$$

(3) $$\begin{array}{c} Specific\;growth\;rate\left(SGR,\%\;day^{1}\right)=100\times \left[ln\left(Final\;individual\;weight\right)-ln\;\left(initial\;individual\;weight\right)\right]/Days, \end{array}$$

(4) $$\begin{array}{c} Condition\;factor\;\left(CF,\%\right)=100\times \left(Final\;individual\;weight/Final\;individual\;weight\;lenght^{3}\right), \end{array}$$

(5) $$\begin{array}{c} Hepatosomatic\;index\;\left(HSI,\%\right)=100\times \left(Wet\;hepatopancreatic\;weight/Final\;individual\;weight\right). \end{array}$$

### 2.5. Nutrient Proximate Chemical Composition and Amino Acids Analysis

The measurements of the proximate chemical composition of all diets and whole crayfish were detected according to the previous procedure [20]. Crude protein was analyzed by the Dumas combustion method (Elementar rapid N exceed, Germany). Moisture was calculated gravimetrically by drying at 105°C in an oven for 24 hr. Lipid content was analyzed by ether extraction. Ash content was tested using a SX2-4-10N furnace machine.

Amino acid content analysis was tested using the HPLC method. The freeze-dried diets and muscle were hydrolyzed with 6 N HCI in a 110°C oven for 24 hr. Phenyl isothiocyanate was used to synthesize phenyl thiocarbamate via precolumn derivatization of amino acids. To prevent oxidation of methionine, 0.1% phenol was used during acid digestion. An automatic analyzer (Biochrom 20, England) was used for the amino acid contents of the hydrolysate quantification.

### 2.6. Digestive Enzymes Activities Analysis

After pretreatment and centrifugation, the homogenate supernatant of samples was carefully collected and frozen in an ultra-low-temperature refrigerator until analysis. The amylase, pepsin, trypsin, and lipase were analyzed using commercial kits from Nanjing Jiancheng Bioengineering Institute (Codes C016, A080, A080, A054), and the enzyme activities were assayed according to the previous procedure [21, 22].

### 2.7. Antioxidant Capacity

The hemolymph of two crayfish in each replicate was collected and pretreat all samples. After that, the hemolymph supernatant was used for analysis of total antioxidant capacity (T-AOC), alanine aminotransferase (ALT), aspartate aminotransferase (AST), glutathione peroxidase (GPx), superoxide dismutase (SOD), and malondialdehyde (MDA), which were measured by commercial kits (Codes. A015, C009, C010, A005, A001, and A003; Jiancheng, Nanjing, China). The alkaline phosphatase (AKP), acid phosphatase (ACP) in the hepatopancreas were also determined by commercial kits (Codes A059, A060; Jiancheng, Nanjing, China). T-AOC, GPx, SOD, MDA, AST, ALT, ACP, and AKP in samples were tested according to the previous method, respectively [23–28]. All enzyme activities and MDA were determined by absorbance colorimetry. The specific wavelengths of T-AOC, GPx, SOD, MDA, AST, and ALT are 593, 412, 450, 532, 510, and 510 nm, respectively. ACP and AKP utilize disodium phenyl phosphate as a substrate to produce red quinone derivatives, and the enzyme activity was calculated by measuring the rate of increase in absorbance at 520 nm. Total protein content was detected based on the Bradford [29] method via a commercial kit with Code A045 (Jiancheng, Nanjing, China).

### 2.8. Histological Analysis

The intestines of three crayfish in each treatment were used to histological observation. Midintestinal samples were quickly removed and fixed for 24 hr in 4% paraformaldehyde. After undergoing a dehydration process in ethanol with graded levels and hyalinization in xylol, the treated intestine was embedded in paraffin wax. The 5 *μ*m tissue slices were stained with hematoxylin–eosin. The observation and photographs of paraffin sections were performed by a microscope (ECLIPSE 200, Nikon, Japan).

### 2.9. Crayfish Intestine Microbiome Sequencing Analysis

The total intestinal DNA was independently isolated via a commercial kit (Omega, Norcross, USA) based on the extraction guidelines. After quantity and quality detection, the V3-V4 region of 16S rRNA was amplified by PCR using 338F (5′- ACTCCTACGGGAGGCAGCA-3′) and 806R (5′- GGACTACHVGGGTWTCTAAT-3′). PCR product was purified and recycled using 2% agarose gel, and then, purified products content was quantified using Quantus^TM^ Fluorometer (Promega, USA). Purified amplicons were sequenced using the Illumina MiSeq PE300 platform (Majorbio, Shanghai, China). The raw data are available in NCBI (accession: SRP310046).

OTUs were clustered with a similarity threshold of 97% using UPARASE (Version 7.0.1), and then, the representative sequence was annotated using RDP classifier (Version 2.2) [30]. Alpha diversity indices (Ace index, Chao1 index, Simpson index, and Shannon index) were calculated by using QIIME (Version 1.9) and utilized *t* tests to determine the difference between control and each treatment. Overall difference in bacterial community was evaluated by nonmetric multidimensional scaling (NMDS) [31]. The statistical difference in each group was determined by using LEfSe. PICRUSt [32] was used to predict the microbial function by using 16S data and metagenomic data, and then, predicted pathways were predicted according to the KEGG catalogue. Gut microbiota interspecies interactions among the dominant genera, each with abundances in the top 30 for the three treatments, were calculated using Mothur. The interspecies network was visually presented and calculated via Gephi (Rho > 0.5 and *P* < 0.05).

### 2.10. Statistical Analysis

All raw data are depicted as Mean ± SE. All data obtained from the present study were analyzed via ANOVA using SPSS program (IBM, version 25.0). Tukey's test was applied to compare the significance, when significant difference was found among experimental groups (*P* < 0.05).

## 3. Results

### 3.1. Growth Indices

The WG and SGR of crayfish significantly increased with 0.4% dietary lysine and threonine supplementation (*P* < 0.05; Table 3). The survival, HSI, and CF of crayfish were not significantly affected by dietary lysine and threonine supplementation (*P* > 0.05; Table 3).

### 3.2. The Proximate Composition and Amino Acids Profile

After 56 days feeding trial, different dietary lysine and threonine supplementation had no significant influence on the proximate composition of whole crayfish (*P* > 0.05; Table 4). Moreover, the percentage concentrations of histidine in the groups fed with L0.2, L0.4, and T0.2 diets were markedly lower than crayfish fed with control diet (*P* > 0.05; Table 5).

### 3.3. Digestive Enzyme Activity

**Figure 1:**
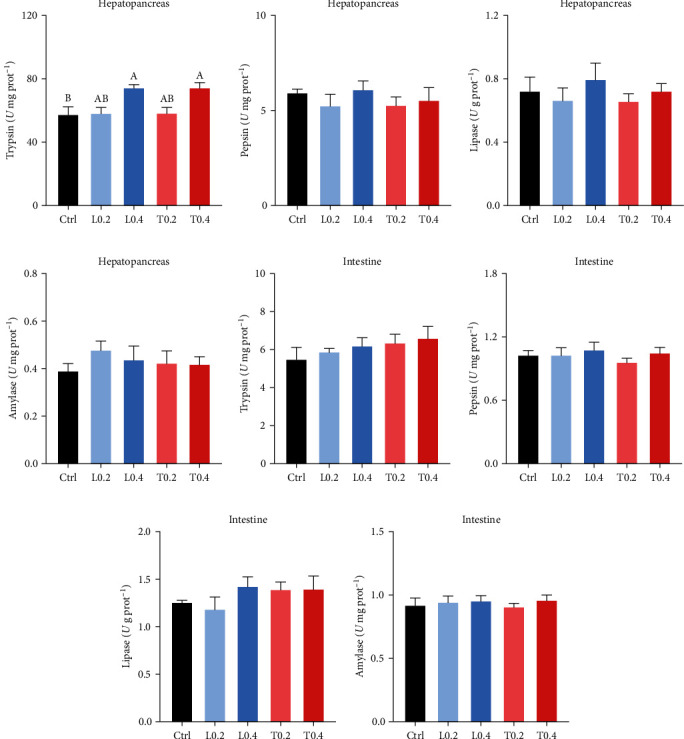
The digestive enzyme activity in the Hepatopancreas and Intestine of *C. quadricarinatus* fed with five experimental diets. Trypsin (a), pepsin (b), lipase (c), and amylase (d) in Hepatopancreas. Trypsin (e), pepsin (f), lipase (g), and amylase (h) in Intestine. Ctrl means control diet without lysine and threonine supplementation; L0.2 and L0.4 means experimental diets with 0.2% and 0.4% lysine, respectively; T0.2 and T0.4 means experimental diets with 0.2% and 0.4% threonine, respectively. Mean values with similar letters are not significant difference (*P* > 0.05; a ＞ b ＞ c). Data are expressed as mean ± SE (*n* = 8).

The activity of digestive enzymes in the hepatopancreas and intestine of crayfish among all treatments is shown in Figure 1. The hepatopancreas trypsin activity in the L0.4 and T0.4 groups was increased significantly compared crayfish in the control (*P* < 0.05; Figure 1(a)). No significant difference in the pepsin, lipase, amylase, and intestinal trypsin activities was detected among all groups (*P* > 0.05; Figures 1(b)–1(h).

### 3.4. Antioxidant Status

**Figure 2:**
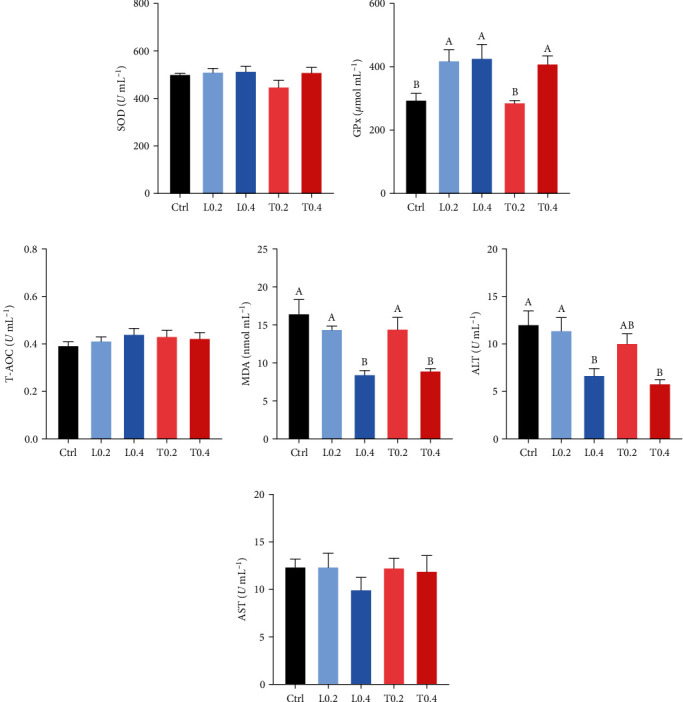
The antioxidant capacity in serum of *C. quadricarinatus* fed with five experimental diets. SOD (a), GPx (b), T-AOC (c), MDA (d), ALT (e), and AST (f) in serum. Ctrl means control diet without lysine and threonine supplementation; L0.2 and L0.4 means experimental diets with 0.2% and 0.4% lysine, respectively; T0.2 and T0.4 means experimental diets with 0.2% and 0.4% threonine, respectively. Mean values with similar letters are not significant difference (*P* > 0.05; a ＞ b ＞ c). Data are expressed as mean ± SE (*n* = 8). Superoxide dismutase (SOD), glutathione peroxidase (GPx), total antioxidant capacity (T-AOC), malondialdehyde (MDA), alanine aminotransferase (ALT), aspartate aminotransferase (AST).

**Figure 3:**
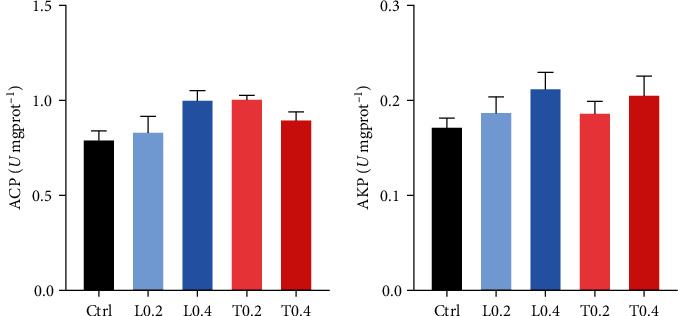
The hepatopancreas ACP (a) and AKP (b) activities of *C. quadricarinatus* fed with five experimental diets. Ctrl means control diet without lysine and threonine supplementation; L0.2 and L0.4 means experimental diets with 0.2% and 0.4% lysine, respectively; T0.2 and T0.4 means experimental diets with 0.2% and 0.4% threonine, respectively. Mean values with similar letters are not significant difference (*P* > 0.05; a ＞ b ＞ c). Data are expressed as mean ± SE (*n* = 8). Alkaline phosphatase (AKP), acid phosphatase (ACP).

In terms of the serum SOD, T-AOC, and AST, no significant difference was detected among all groups (*P* > 0.05; Figures 2(a), 2(c) and 2(f)). The activities of GPx in the L0.4 and T0.4 groups were significantly higher compared to the control (*P* < 0.05; Figure 2(b)). Moreover, compared to other groups, MDA content in the L0.4 and T0.4 treatments was markedly decreased (*P* < 0.05; Figure 2(d)). Similarly, no significant difference of ACP and AKP in hepatopancreas was observed among experimental treatments (*P* > 0.05; Figures 3(a) and 3(b)).

### 3.5. Intestine Histology

**Figure 4:**
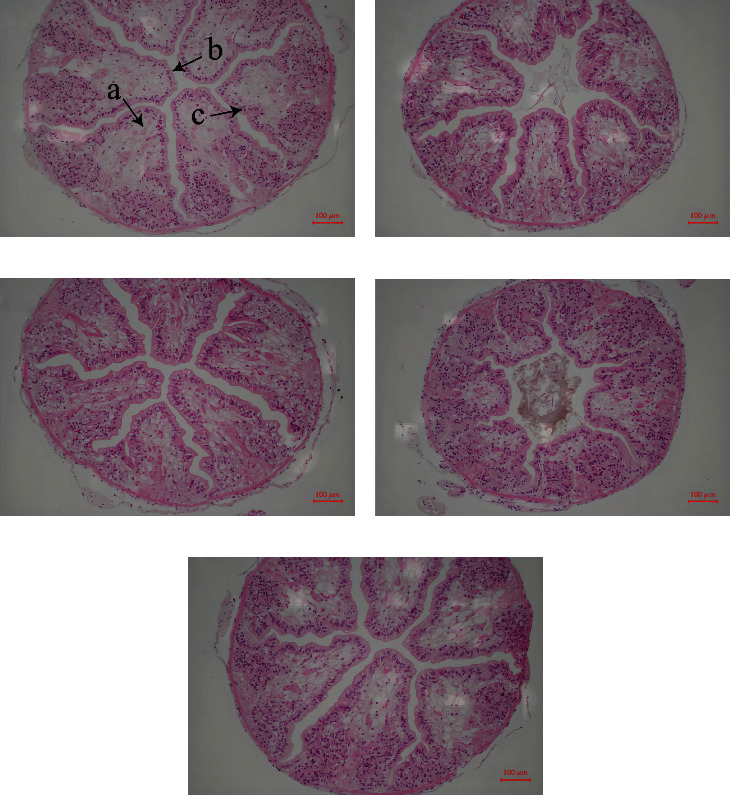
Intestinal histology of *C. quadricarinatus* fed with five experimental diets. (a) Ctrl means control diet without lysine and threonine supplementation; (b) and (c) L0.2 and L0.4 mean experimental diets with 0.2% and 0.4% lysine, respectively; (d) and (e) T0.2 and T0.4 mean experimental diets with 0.2% and 0.4% threonine, respectively. The magnification was 40x. Arrow (a) represents brush border, (b) epithelium, (c) nuclei.

From histological observation of the intestine, intestinal folds and epithelial cells remained complete among all groups (Figure 4).

### 3.6. Difference Analysis of Gut Microbiota

**Figure 5:**
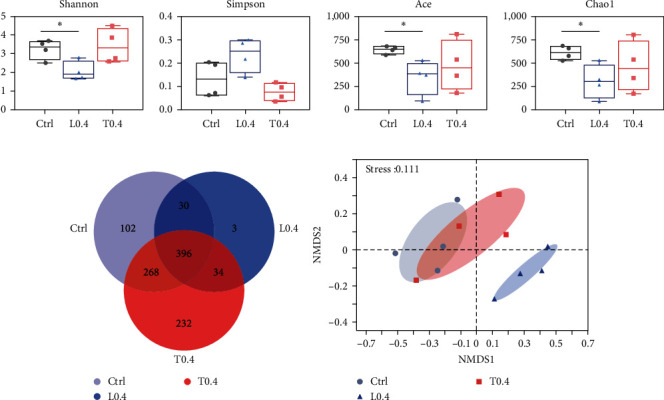
The *α*-diversity indices of intestinal microbiota. (a) Box plots depict Shannon, Simpson, Ace, and Chao1 index.  ^*∗*^*P* < 0.05. Venn diagram (b) and NMDS analysis (c).

A total of 509,362 raw sequences were obtained from 12 gut samples in three groups, with an average of 42,447 sequences per sample. The alpha diversity analysis is presented in Figure 5. Shannon, Ace, and Chao1 indices in crayfish fed the diet with 0.4% lysine supplementation were markedly lower than crayfish fed the control (*P* < 0.05; Figure 5(a)). Ctrl, L0.4, and T0.4 groups contained 102, 3, and 232 OTUs, respectively, and the number of OTUs shared by the three groups is 396 (Figure 5(b)).

For NMDS analysis, the microbiota in the L0.4 group was clearly separated from those in the control and T0.4 groups (Figure 5(c)).

**Figure 6:**
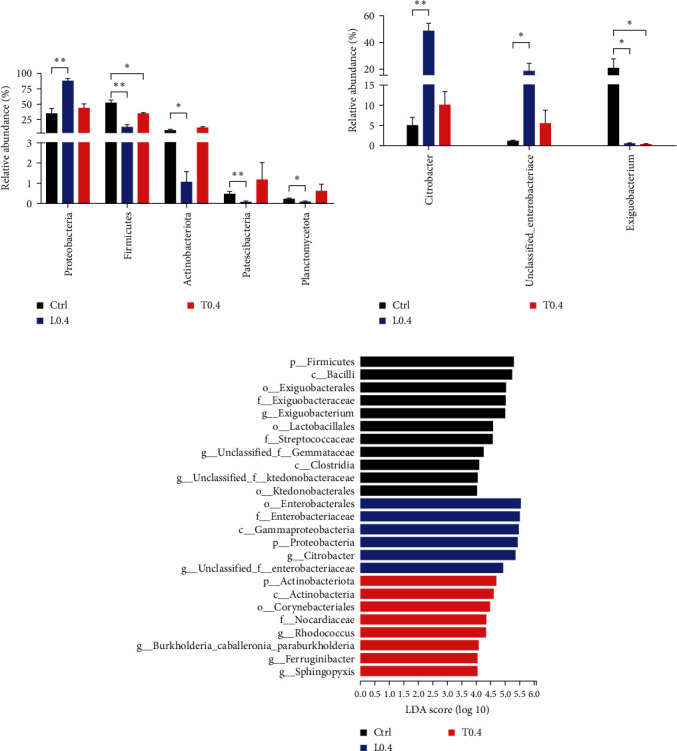
Differential bacterial relative abundance at the phylum level (a), differential bacterial relative abundance at the genus level (b) and bacterial taxa differentially displayed in three groups identified by LEfSe using LDA score threshold of >4 (c).

At phylum level, the abundance of *Firmicutes*, *Actinobacteriota*, *Patescibacteria*, and *Planctomycetota* in the L0.4 group was significantly lower compared with the control (*P* < 0.05; Figure 6(a)). Moreover, the abundance of *Proteobacteria* in crayfish fed the L0.4 diet was significantly higher than in the control (*P* < 0.05). Meanwhile, the abundance of *Firmicutes* in the T0.4 group was also markedly lower compared to those crayfish fed the control (*P* < 0.05). In addition, the abundance of genera revealed that L0.4 diets significantly increased *Cirrobacter* and *Unclassified Enterobacteriace* in crayfish compared with the control (*P* < 0.05; Figure 6(b)). However, by feeding L0.4 and T0.4 diets for a 56-day trial, the abundance of *Exiguobacteriace* was markedly lower compared to those crayfish fed with the control diet (*P* < 0.05; Figure 6(b)). LEfSe analysis showed that eight bacteria genera enriched in the T0.4 group, and only six bacteria genera enriched in the L0.4 group (Figure 6(c)).

### 3.7. Gut Microbiota Functional Prediction

The function of the gut microbiota is shown in Table 6. The abundance of two pathways significantly changed in the KEGG Level 3 pathway analysis. The abundance of fatty acid elongation in mitochondria and apoptosis in crayfish fed the L0.4 diet exhibited a markedly decreased compared to crayfish fed the control diet (*P* < 0.05).

### 3.8. Gut Microbiota Interspecies Interaction Analysis

**Figure 7:**
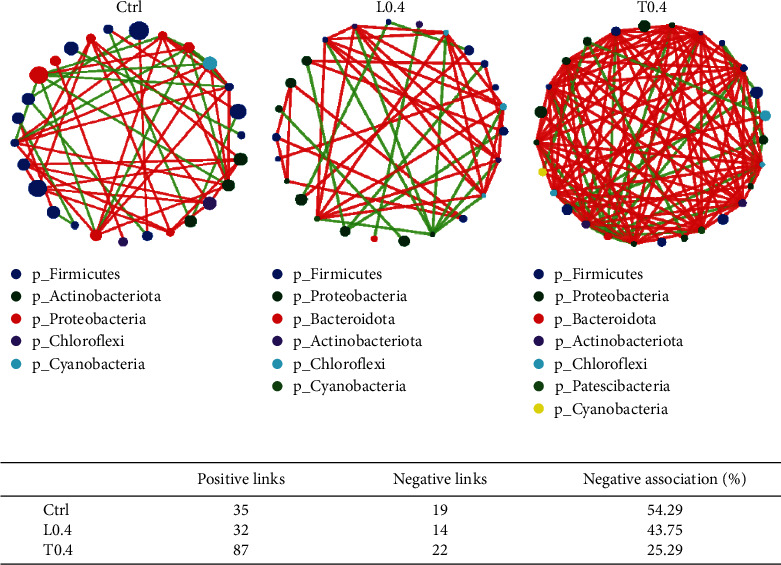
Interspecies interaction network of intestinal microbiota for C. quadricarinatus fed diets with the Ctrl, L0.4 and T0.4 diets (a). Each node represents a genus, with node colors indicating bacteria at the phylum level. A green line represents positive interaction, and a red line represents negative interaction. The number and ratio of interspecies positive and negative interactions in the ecological network (b).

Interspecies interaction networks are shown in Figure 7. Compared to crayfish fed with the control, the crayfish in the L0.4 group had fewer negative links, and the T0.4 group showed more negative links (Figure 7(b)). A tighter interspecies interaction network and more correlation links were found in the intestines when crayfish were fed the T0.4 diet (Figure 7(a) and 7(b)).

## 4. Discussion

Dietary threonine and lysine supplementation significantly enhance the growth parameters of *C. quadricarinatus*, which is in agreement with the results from *Marsupenaeus japonicus*, *Megalobrama amblycephala*, and *Myxocyprinus asiaticus* [33–35]. Lysine and threonine are considered as essential amino acids in crustacean nutritional requirements that cannot be biosynthesized and must be obtained through food. However, previous studies have reported that dietary crystalline amino acid excess may cause growth inhibition of *Cirrhinus mrigala* and *Labeo rohita* [36, 37]. In this study, the whole crayfish composition, lysine and threonine deposition in muscle were not significantly affected by dietary lysine and threonine levels. This is consistent with the previous studies on *Litopenaeus vannamei* and *Rhamdia voulezi* [38, 39]. Excess of certain amino acids may result in deamination and inhibition of amino acids, which have further led to toxic effects on aquatic animals [40–42]. The histidine content in the crayfish tail muscle in the lysine supplementation groups was markedly reduced when compared with the control. Similarly, previous results revealed that dietary lysine and threonine content excess led to other amino acids decrease in the body of animals [33, 43].

Nutrients are digested mainly by digestive enzymes, and enzyme activity can also reflect digestive capacity. Researchers have also found that supplementation with exogenous amino acids in feed could promote growth performance by increasing digestive enzyme activity [35]. The T0.4 and L0.4 diets significantly increased hepatopancreatic trypsin activity, confirming a positive correlation between WG and digestive enzyme activity. Furthermore, whole-body composition was not affected by fed with all diets, in agreement with prior research on *Megalobrama amblycephala* [34].

ALT and AST are always regarded as essential cellular and hepatopancreatic damage indicators [44]. When the serum ALT and AST activities increase, it implies that hepatopancreas function has been damaged [45]. The control group exhibited the highest transaminase values, suggesting that the dietary inclusion of lysine and threonine can mitigate hepatopancreatic damage. The AKP and ACP in serum are the two essential enzymes in the immunity of aquatic animals [46]. AKP and ACP activities in serum increased with dietary lysine and threonine supplementation. ACP and AKP are both crucial lysosomal enzymes in aquatic animals, and their high activity indicates enhancement of immune function. These important enzymes participate in the degradation of nutrients and phagocytosis [47].

Antioxidant enzymes, including SOD and GPx, are the first line of the antioxidant enzymatic defense barrier to eliminate intracellular ROS production to reduce oxidative damage to the organisms [48]. Serum MDA concentration is commonly utilized as an indicator of protein and lipid peroxidation, and its content is usually inversely correlated with antioxidant enzyme activity [49]. Antioxidant enzyme activity can represent the level of antioxidant capacity. This phenomenon was observed in the current study; crayfish fed with the T0.4 and L0.4 diets exhibited higher GPx activity and lower MDA concentration compared to the control group. Dietary threonine deficiency increases ROS and MDA and decreases antioxidant enzyme activities of *Ctenopharyngodon idella* by regulating the NFE2-related Factor 2 signaling pathway [50]. Similar results were observed with increased overall antioxidant capacity when supplemented with lysine and threonine in diets [34, 38].

Animal growth and development are mainly associated with digestion and absorption ability. Normal intestinal structure in animals plays a crucial role in the digestion and absorption of nutrients [51]. So far, no study has evaluated the effects of dietary lysine and threonine supplementations on the intestinal structure of crayfish. The intestinal structure of crayfish was not affected by supplementing 0.4% lysine or threonine in the diet. Similar studies demonstrated that dietary lysine and threonine supplementations do not affect the intestinal structure of broilers [52]. However, dietary lysine deficiency led to the separation of head kidney endothelial cells of *Ctenopharyngodon idella* and hemorrhage of spleen [53].

Maintaining a stable intestinal flora structure is crucial for both immune and digestive functions [54]. In this study, L0.4 diets significantly decreased Shannon, Ace, and Chao 1 indices compared to the control. However, microbial diversity plays a crucial role in intestinal function but depends on the existence of beneficial bacteria rather than variety itself [55]. According to many studies, *Proteobacteria*, *Firmicutes*, and *Actinobacteriota*, as the most dominant phylum, were observed in the crustacean intestine [56]. At the phylum level, a similar composition of gut microbiota was found in all groups. A previous study showed that elevated relative abundance of *Proteobacteria* results in intestinal microbiota disorders [57]. Furthermore, *Firmicutes* and *Actinobacteriota* are probiotics that are used to keep the intestinal microbiota stable for disease resistance [58, 59]. In the present study, *Proteobacteria* was significantly increased in the L0.4 group, while *Firmicutes* and *Actinobacteriota* were significantly decreased in the L0.4 group. *Cirrobacter* and *Enterobacteriace* are well known as the opportunistic pathogen [60, 61]. LefEs analysis indicated that *Proteobacteria*, *Enterobacteriace*, and *Cirrobacter* were dominant in the L0.4 group, while *Actinobacteriota* and *Rhodococcus* were dominant in the T0.4 group.

The predictive analysis of gut microbial function is vital to immunity. Apoptosis process is used to remove infected, unneeded, and deleterious cells in the body [62]. According to a previous study, apoptosis also can modulate the level of immune response and establish immune memory [63]. Therefore, the apoptosis in cellular processes was decreased significantly in the intestinal function of the L0.4 group compared to the control, which may have a negative impact on the growth and immunity of crayfish. Thus, the intestinal microbial composition and bacterial function are regulated by dietary lysine and threonine.

The relationships of cooperation and competition exist in different intestinal microbiota, and various species interact with each other to establish a complex ecological network [64]. The positive and negative links were used to demonstrate the cooperative and competitive relationship among the different bacterial communities [65]. The stable structure of intestinal microbiota depends on the high ratio of negative links [66]. A large number of interactions within the microbial interaction network can also enhance the stability of the microbial community structure [67]. A similar result was found in the T0.4 group. According to a previous study, as the diversity and abundance of gut microbiota increase, the microbial correlation network becomes denser, and the microbial structure becomes more stable [68]. The reason for the instability of the microbial structure in the L0.4 group may be due to the significant decrease in the alpha indices. Dietary lysine and threonine supplementation effects on gut microbiota architecture and interaction networks are unclear in *C. quadricarinatus* and require further study.

## 5. Conclusion

The 0.4% group had better growth performance compared to the control and 0.2% groups. However, the 0.4% lysine group resulted in lower gut microbiota abundance and an unstable intestinal structure. Moreover, the inclusion of 0.4% threonine supplementation in a vegetable-based diet for *C. quadricarinatus* significantly enhances growth performance, digestibility, antioxidant statue, and stable intestinal flora structure. Moreover, further research should consider the interaction of essential amino acid supplementation in the diet of *C. quadricarinatus* for metabolic mechanism.

## Figures and Tables

**Table 1:** Ingredients composition and nutrient content of experimental diets (g/kg dry matter basis).

Ingredients (%)	Treatments				
	Ctrl	L0.2	L0.4	T0.2	T0.4
Fish meal	245	245	245	245	245
Soybean meal	187	187	187	187	187
Cottonseed meal	201	201	201	201	201
Wheat starch	180	180	180	180	180
Soybean oil	50	50	50	50	50
Lysine^1^	0	2	4	0	0
Threonine^1^	0	0	0	2	4
Cholesterol^1^	5	5	5	5	5
Lecithin^2^	10	10	10	10	10
Vitamin premix^3^	20	20	20	20	20
Mineral premix^4^	20	20	20	20	20
Choline chloride^1^	10	10	10	10	10
Sodium carboxymethylcellulose	25	25	25	25	25
Cellulose	47	45	43	45	43
Total	1,000	1,000	1,000	1,000	1,000
Proximate composition (g/kg)					
Crude protein	369.5	371.6	372.5	372.7	374.5
Crude lipid	95.8	96.2	95.6	96.0	95.9
Ash	88.5	90.9	89.0	89.7	90.1
Moisture	84.9	83.2	84.5	85.4	84.6

^1^Obtained from Sangon Biotech, Ltd., Shanghai, China. ^2^Obtained from Shanghai Taiwei, Ltd., Shanghai, China. ^3^Reference as Jiang et al. [8]. ^4^Reference as Jiang et al. [8]. Ctrl means control diet without lysine and threonine supplementation; L0.2 and L0.4 mean experimental diets with 0.2% and 0.4% lysine, respectively; T0.2 and T0.4 mean experimental diets with 0.2% and 0.4% threonine, respectively.

**Table 2:** Amino acid composition of the experimental diets (g/100 g dry matter).

Amino acid	Treatments				
	Ctrl	L0.2	L0.4	T0.2	T0.4
Essential amino acid					
Arginine (Arg)	3.29 ± 0.01	3.28 ± 0.01	3.28 ± 0.01	3.29 ± 0.01	3.29 ± 0.04
Histidine (His)	0.75 ± 0.01	0.73 ± 0.01	0.73 ± 0.01	0.74 ± 0.01	0.74 ± 0.01
Isoleucine (Ile)	1.44 ± 0.01	1.44 ± 0.01	1.44 ± 0.02	1.45 ± 0.02	1.44 ± 0.01
Leucine (Leu)	2.5 ± 0.02	2.44 ± 0.01	2.45 ± 0.04	2.51 ± 0.02	2.58 ± 0.07
Lysine (Lys)	1.65 ± 0.01^c^	1.73 ± 0.01^b^	2.02 ± 0.01^a^	1.67 ± 0.01^bc^	1.68 ± 0.02^b^
Methionine (Met)	0.49 ± 0.04	0.53 ± 0.01	0.51 ± 0.01	0.51 ± 0.03	0.53 ± 0.01
Phenylalanine (Phe)	1.84 ± 0.01	1.83 ± 0.01	1.84 ± 0.01	1.84 ± 0.01	1.84 ± 0.01
Threonine (Thr)	1.38 ± 0.01^c^	1.34 ± 0.01^c^	1.35 ± 0.02^c^	1.49 ± 0.01^b^	1.83 ± 0.05^a^
Valine (Val)	1.89 ± 0.02	1.85 ± 0.01	1.86 ± 0.03	1.87 ± 0.03	1.89 ± 0.02
Nonessential amino acid					
Alanine (Ala)	1.56 ± 0.03	1.56 ± 0.01	1.56 ± 0.02	1.57 ± 0.01	1.57 ± 0.01
Aspartic acid (Asp)	3.32 ± 0.02	3.28 ± 0.02	3.29 ± 0.03	3.33 ± 0.02	3.38 ± 0.06
Glutamic acid (Glu)	6.17 ± 0.02	6.12 ± 0.04	6.10 ± 0.10	6.16 ± 0.01	6.10 ± 0.01
Glycine (Gly)	2.46 ± 0.02	2.44 ± 0.03	2.44 ± 0.03	2.46 ± 0.02	2.45 ± 0.01
Proline (Pro)	1.86 ± 0.02	1.87 ± 0.02	1.86 ± 0.03	1.88 ± 0.04	1.86 ± 0.07
Serine (Ser)	2.17 ± 0.05	2.17 ± 0.02	2.16 ± 0.04	2.15 ± 0.01	2.16 ± 0.01
Tyrosine (Tyr)	1.46 ± 0.01	1.44 ± 0.03	1.45 ± 0.02	1.45 ± 0.02	1.47 ± 0.01
Total Essential amino acid	14.99 ± 0.07^b^	14.95 ± 0.04^b^	15.26 ± 0.14^ab^	15.14 ± 0.05^b^	15.62 ± 0.14^a^
Total nonessential amino acid	19.00 ± 0.12	18.89 ± 0.08	18.84 ± 0.19	19.01 ± 0.04	18.98 ± 0.03
Total amino acid	34.00 ± 0.19	33.83 ± 0.11	34.10 ± 0.30	34.15 ± 0.09	34.60 ± 0.17

Mean values with similar letters are not significant difference (*P*  > 0.05 a ＞ b ＞ c). Data expressed as mean ± SE (*n* = 4). Ctrl means control diet without lysine and threonine supplementation; L0.2 and L0.4 mean experimental diets with 0.2% and 0.4% lysine, respectively; T0.2 and T0.4 mean experimental diets with 0.2% and 0.4% threonine, respectively.

**Table 3:** Growth parameters of *C. quadricarinatus* fed diets with different levels of lysine and threonine for 8 weeks.

Parameters	Treatments				
	Ctrl	L0.2	L0.4	T0.2	T0.4
Initial weight (g)	10.69 ± 0.31	11.47 ± 0.44	11.58 ± 0.35	11.94 ± 0.41	11.91 ± 0.34
Final weight (g)	21.75 ± 0.94^c^	23.23 ± 1.07^bc^	26.2 ± 0.73^ab^	25.38 ± 0.75^abc^	27.59 ± 1.07^a^
WG (%)	103.62 ± 8.78^b^	102.57 ± 9.37^b^	126.26 ± 6.33^a^	112.58 ± 6.31^ab^	131.67 ± 9.00^a^
SGR (%)	19.62 ± 1.83^b^	20.29 ± 1.92^b^	27.51 ± 1.63^a^	23.81 ± 1.44^ab^	28.77 ± 1.58^a^
HSI (%)	7.26 ± 0.36	6.97 ± 0.25	7.03 ± 0.53	7.84 ± 0.50	6.67 ± 0.37
CF (%)	2.09 ± 0.06	2.00 ± 0.05	2.18 ± 0.04	2.02 ± 0.06	2.16 ± 0.19
Survival (%)	73.54 ± 2.42	74.75 ± 1.30	74.35 ± 2.53	75.05 ± 2.41	75.25 ± 2.37

Mean values with similar letters are not significant difference (*P* > 0.05; a ＞ b ＞ c). Data expressed as mean ± SE (*n* = 8). Ctrl means control diet without lysine and threonine supplementation; L0.2 and L0.4 mean experimental diets with 0.2% and 0.4% lysine, respectively; T0.2 and T0.4 mean experimental diets with 0.2% and 0.4% threonine, respectively. WG, weight gain; SGR, specific growth rate; HSI, hepatosomatic index; CF, condition factor.

**Table 4:** The whole-body proximate composition of *C. quadricarinatus* fed diets with different levels of lysine and threonine (g/kg wet weight).

Parameters	Treatments				
	Ctrl	L0.2	L0.4	T0.2	T0.4
Crude protein	127.41 ± 1.19	127.72 ± 2.20	128.13 ± 3.55	127.14 ± 3.03	128.58 ± 3.25
Crude lipid	26.61 ± 2.54	27.36 ± 4.36	26.82 ± 1.79	27.52 ± 1.95	26.37 ± 1.68
Moisture	723.56 ± 5.48	706.9 ± 7.60	724.8 ± 10.70	709.9 ± 6.90	736.40 ± 7.40
Ash	80.16 ± 3.20	85.61 ± 3.36	81.51 ± 3.12	83.76 ± 2.00	84.27 ± 2.90

Mean values with similar letters are not significant difference (*P* > 0.05; a ＞ b ＞ c). Data expressed as mean ± SE (*n* = 3). Ctrl means control diet without lysine and threonine supplementation; L0.2 and L0.4 mean experimental diets with 0.2% and 0.4% lysine, respectively; T0.2 and T0.4 mean experimental diets with 0.2% and 0.4% threonine, respectively.

**Table 5:** Amino acid composition in muscle of *C. quadricarinatus* (g/100 g dry matter).

Amino acid	Treatments				
	Ctrl	L0.2	L0.4	T0.2	T0.4
Essential amino acid					
Arginine (Arg)	10.55 ± 0.02	10.27 ± 0.17	9.98 ± 0.33	10.47 ± 0.23	10.47 ± 0.13
Histidine (His)	2.59 ± 0.03^a^	2.31 ± 0.05^b^	2.31 ± 0.02^b^	2.29 ± 0.04^b^	2.47 ± 0.02^a^
Isoleucine (Ile)	3.79 ± 0.10	3.8 ± 0.05	3.8 ± 0.05	3.82 ± 0.04	3.49 ± 0.31
Leucine (Leu)	6.90 ± 0.09	6.67 ± 0.1	6.64 ± 0.1	6.69 ± 0.07	6.68 ± 0.02
Lysine (Lys)	7.50 ± 0.18	7.43 ± 0.1	7.40 ± 0.15	7.42 ± 0.10	7.34 ± 0.01
Methionine (Met)	2.14 ± 0.08	1.71 ± 0.27	1.63 ± 0.38	1.71 ± 0.24	2.13 ± 0.03
Phenylalanine (Phe)	3.67 ± 0.06	3.63 ± 0.05	3.64 ± 0.05	3.64 ± 0.04	3.64 ± 0.01
Threonine (Thr)	3.44 ± 0.06	3.44 ± 0.06	3.44 ± 0.03	3.52 ± 0.05	3.44 ± 0.01
Valine (Val)	4.05 ± 0.04	4.02 ± 0.06	4.00 ± 0.04	4.04 ± 0.05	4.00 ± 0.01
Nonessential amino acid					
Alanine (Ala)	4.67 ± 0.11	4.57 ± 0.07	4.55 ± 0.11	4.65 ± 0.10	4.70 ± 0.04
Aspartic acid (Asp)	9.07 ± 0.04	8.83 ± 0.11	8.83 ± 0.1	8.88 ± 0.09	8.84 ± 0.01
Glutamic acid (Glu)	14.47 ± 0.12	14.27 ± 0.25	14.22 ± 0.19	14.70 ± 0.22	14.48 ± 0.07
Glycine (Gly)	4.33 ± 0.40	3.66 ± 0.18	3.62 ± 0.26	4.00 ± 0.14	4.28 ± 0.23
Proline (Pro)	2.72 ± 0.05	2.85 ± 0.01	2.84 ± 0.02	2.85 ± 0.1	2.78 ± 0.03
Serine (Ser)	3.64 ± 0.06	3.57 ± 0.05	3.57 ± 0.07	3.45 ± 0.04	3.47 ± 0.01
Tyrosine (Tyr)	3.48 ± 0.06	3.43 ± 0.05	3.44 ± 0.04	3.70 ± 0.12	3.62 ± 0.01
Total essential amino acid	44.63 ± 0.16^a^	42.74 ± 0.40^ab^	42.60 ± 1.18^b^	43.66 ± 0.59^ab^	43.41 ± 0.30^ab^
Total nonessential amino acid	42.36 ± 0.43	42.05 ± 0.51	42.03 ± 0.74	43.47 ± 0.61	42.82 ± 0.46
Total amino acid	87.00 ± 0.58	84.78 ± 0.87	84.64 ± 1.92	87.13 ± 1.1	86.23 ± 0.74

Mean values with similar letters are not significant difference (*P* > 0.05 a ＞ b ＞ c). Data expressed as mean ± SE (*n* = 4). Ctrl means control diet without lysine and threonine supplementation; L0.2 and L0.4 mean experimental diets with 0.2% and 0.4% lysine, respectively; T0.2 and T0.4 mean experimental diets with 0.2% and 0.4% threonine, respectively.

**Table 6:** The relative abundance of microbial-mediated function *C. quadricarinatus*.

Level	KEGG pathway	Ctrl (%)	L0.4 (%)	T0.4(%)	*P* value	*P* value
					Ctrl vs. L0.4	Ctrl vs. T0.4
3	Fatty acid elongation in mitochondria	2.07E-7	0	1.91E-7	0.001	0.689
3	Apoptosis	1.24E-4	1.79E-5	1.05E-4	0.038	0.680

## Data Availability

Data will be made available on request.
